# Vector uncoating limits adeno-associated viral vector-mediated transduction of human dendritic cells and vector immunogenicity

**DOI:** 10.1038/s41598-019-40071-1

**Published:** 2019-03-06

**Authors:** Axel Rossi, Léa Dupaty, Ludovic Aillot, Liang Zhang, Célia Gallien, Michael Hallek, Margarete Odenthal, Sahil Adriouch, Anna Salvetti, Hildegard Büning

**Affiliations:** 1 International Center for Research in Infectiology (CIRI), INSERM U1111 – Université claude Bernard Lyon 1, CNRS UMR5308, Ecole Normale Supérieur de Lyon, Université de Lyon, Lyon, France; 2 Institute of Experimental Hematology, Hannover Medical School, Hannover, Germany; 3 Normandie Univ, UNIROUEN, INSERM, U1234, Physiopathologie et biothérapies des maladies inflammatoires et autoimmunes (PANTHER), 76000, Rouen, France; 4 Center for Molecular Medicine Cologne (CMMC), University of Cologne, Cologne, Germany; 5 Clinic I of Internal Medicine, University Hospital Cologne, Cologne, Germany; 6 Institute of Pathology, University Hospital Cologne, Cologne, Germany; 7 German Center for Infection Research (DZIF), partner site Hannover-Braunschweig, Hannover, Germany; 8 Present Address: Cancer Research Center of Lyon, INSERM U1052, CNRS UMR5206, Lyon, France

## Abstract

AAV vectors poorly transduce Dendritic cells (DC), a feature invoked to explain AAV’s low immunogenicity. However, the reason for this non-permissiveness remained elusive. Here, we performed an in-depth analysis using human monocyte-derived immature DC (iDC) as model. iDC internalized AAV vectors of various serotypes, but even the most efficient serotype failed to transduce iDC above background. Since AAV vectors reached the cell nucleus, we hypothesized that AAV’s intracellular processing occurs suboptimal. On this basis, we screened an AAV peptide display library for capsid variants more suitable for DC transduction and identified the I/VSS family which transduced DC with efficiencies of up to 38%. This property correlated with an improved vector uncoating. To determine the consequence of this novel feature for AAV’s *in vivo* performance, we engineered one of the lead candidates to express a cytoplasmic form of ovalbumin, a highly immunogenic model antigen, and assayed transduction efficiency as well as immunogenicity. The capsid variant clearly outperformed the parental serotype in muscle transduction and in inducing antigen-specific humoral and T cell responses as well as anti-capsid CD8^+^ T cells. Hence, vector uncoating represents a major barrier hampering AAV vector-mediated transduction of DC and impacts on its use as vaccine platform.

## Introduction

Adeno-associated viral (AAV) vectors have become standard tools for *in vivo* gene transfer^1^. They are composed of a single-stranded (ss) DNA genome packaged within an icosahedral capsid of 20–25 nm in diameter^1^. The 60 viral protein (VP) monomers that assemble into the mature capsid are encoded by a single viral open reading frame (ORF), the *cap* ORF. The two minor VP proteins, VP1 (90 kDa) and VP2 (72 kDa), are N-terminal extensions of the major capsid protein VP3 (60 kDa) but harbor distinct functional domains^2,3^. The common VP3 region - shared by all capsid proteins - forms the basic capsid structure with protrusions at the 3-fold and pores at the 5-fold axis of symmetry^4^. To date, at least 12 AAV serotypes have been isolated from human and non-human primate tissues^1^, which mainly differ in the so-called hypervariable regions of the protrusions^4–11^. These changes in amino acid sequence translate into the serotype-specific tropism that make AAV broadly applicable for gene therapy.

As suggested by pre-clinical *in vivo* studies and now confirmed in human clinical trials, AAV vectors mediate long-term transgene expression in post-mitotic or slowly dividing cells. A critical factor in this regard are unique immunological properties of AAV^12^. More precisely, innate immune responses are only induced at high particle-per-cell ratios and are less pronounced compared to other viral vectors and short-lived^12,13^. Antibodies against the capsid are readily induced upon vector application, and AAV vectors are efficient as vaccines to induce antigen-specific humoral immune responses^14^. However, antigen-specific CD8^+^ T-cell responses appear to be impaired both in intensity and functionality as shown in several mouse and non-human primate models^14^. The latter is a clear advantage for a vector used in gene therapy where induction of transgene-specific T-cell responses results in loss of vector-modified cells^15^.

Generally, T-cell responses are initiated through the capture, processing and presentation of antigens by antigen-presenting cells (APC), and in particular by DC^16–18^. In their immature state, DC are specialized to sense cellular stress, and to detect and engulf pathogens as well as soluble or particulate antigens^18^. DC are rich in pattern recognition receptors (PRR), which, upon pathogen-encounter, induce cell autonomous innate immune signaling leading to cytokine and chemokine secretion, upregulation of adhesion and co-stimulatory molecules, and antigen processing and presentation to CD4^+^ T cells in the context of major histocompatibility complex (MHC) class II molecules. In addition, DC can activate CD8^+^ cytotoxic T cells via MHC class I presentation. The latter route, which is commonly reserved to intracellular antigens, has evolved through a mechanism termed cross-presentation to enable presentation of endocytosed antigens to CD8^+^ T cells. The PRR-induced DC maturation also leads to profound structural and functional reorganization of DC, including changes in uptake routes and profound modifications of the endosomal compartment, including decreased intraluminal pH and increased protease activity^16,19^.

Due to the key function of DC in inducing and shaping adaptive immune responses, the DC-AAV interaction determines vector and transgene-product immunogenicity with a major impact on long-term transgene expression when AAV are used in gene therapy and, conversely, on vaccination efficiency when used as vaccines^14,20^. However, the DC-AAV interaction itself is still poorly characterized. Therefore, we aimed here to study early steps of this interaction and to identify barriers that limit transduction using monocyte-derived immature DC (iDC) as a model system. These analyses revealed that AAV particles are internalized into DC, and that a significant fraction of these particles is also found in the nucleus, suggesting that AAV processing rather than entry or intracellular transport represents the limiting step in transduction. By *in vitro* high-throughput selection screening of our AAV2 peptide display library on iDC we identified a family of capsid variants (I/VSS group) that showed improved transduction of human DC. This property correlated with improved uncoating capacity as compared to the parental serotype. Interestingly, DC maturation considerably influenced the level of transduction, a finding in line with the assumption that efficacy of intracellular processing defines the efficiency of DC transduction by AAV. *In vivo* assays of the AAV capsid variant VSSTSPR, our lead candidate, in an immunogenic transgene model revealed superior transduction efficacy as compared to the parental serotype. In addition, the VSSTSPR variant induced robust antigen-specific antibody and T cell immune responses. Hence, inefficient intracellular processing of AAV particles and, consequently, inefficient vector uncoating are barriers that limit AAV vector-mediated transduction of DC. Such barriers, however, can be overcome by capsid modifications, such as those selected for the VSSTSPR variant, which significantly improved transgene expression levels and help advance this promising vector system for novel applications.

## Results

### AAV vectors are efficiently internalized, but do not transduce human DC

**Figure 1:**
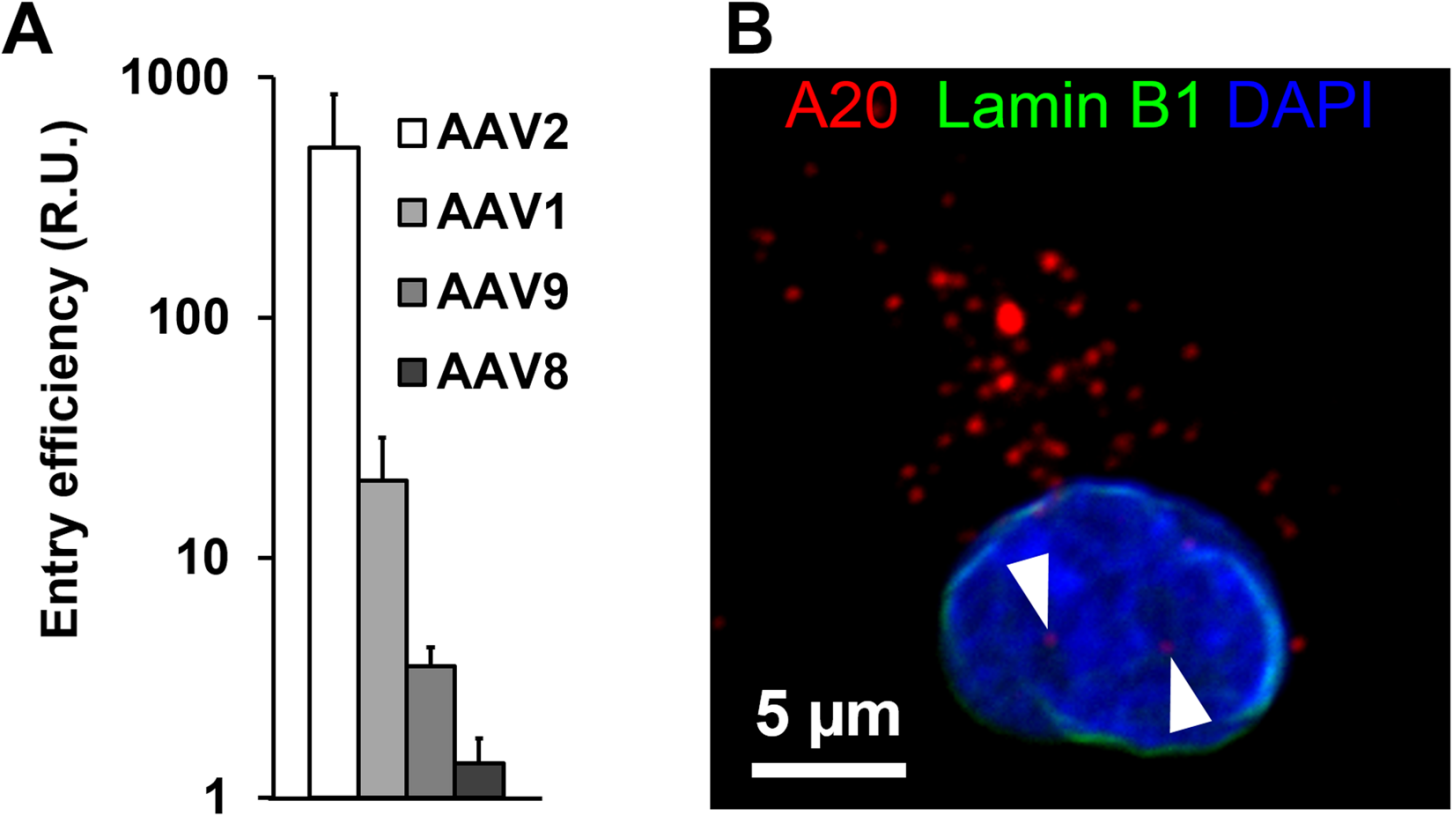
Early steps in iDC transduction by AAV vectors. (**A**) Entry efficiency of AAV vectors derived from different serotypes. iDC were incubated with AAV vectors encoding for enhanced green fluorescent protein (GFP) in single-stranded vector genome conformation at a GOI of 10^4^ for 48 hrs. Cells were harvested by extensive trypsin treatment to remove membrane-bound particles^22^. Total DNA was extracted and analyzed by qPCR using primers specific for GFP (vector genome) and for the host cell gene (β-globin), respectively. Results are presented as the mean GFP-to-β-globin ratio (R.U.). Error bars represent standard deviation (n = 3). (**B**) AAV2 vectors are delivered to the nucleus. iDC were incubated with AAV2 vectors at a GOI of 10^4^ for 7 hrs. Cells were fixed, permeabilized, and incubated with antibodies directed against the AAV capsid (A20) and nuclear Lamin B1, respectively. The nucleus was stained with DAPI. Cells were imaged by confocal microscopy. Arrow heads indicate A20 signals in the nucleus.

Several studies, including ours (Supplementary Fig. S1), indicated inefficient transduction of human DC with AAV even when using vectors at high particle-per-cell ratios (GOI) with a self-complementary vector genome conformation that bypass the need for DNA second-strand synthesis^21^. Aiming to decipher the determinants of this non-permissiveness, we first compared a set of AAV serotypes regarding their cell entry efficiency. Therefore, we incubated iDC with equal amounts of indicated serotype vectors and quantified the number of intracellular vector genomes by qPCR as previously described^22^ (Fig. 1A).

All AAV serotypes were internalized, albeit with remarkable differences in efficiency. Cell entry was most efficient for AAV2 followed by AAV1 and AAV9. For the most efficient serotype, AAV2, we then determined the presence and quantity of AAV vectors in the cytosolic, membrane and nuclear fractions of iDC at 24 and 48 hrs p.i. (Supplementary Fig. S2). For both time points, highest vector genome numbers were measured in the membrane fraction, which also contains endosomes. While at 24 hrs p.i. slightly more vector genomes were detected in the cytosolic compared to the nuclear fraction, the opposite was seen at 48 hrs p.i. Interestingly, however, at both time points substantial amounts of AAV vector genomes were present in the nuclear fraction. To confirm this result by an independent method, we analyzed AAV2-transduced iDC by confocal microscopy (Fig. 1B). Our staining strategy did not allow to distinguish between cytosol and vesicles. Nevertheless, as shown in the representative image, the majority of AAV particles were found in the cytoplasm in discrete foci, suggesting an association with the vesicular system, which is in line with our qPCR-based analyses. Of particular importance, the microscopic analyses also confirmed the presence of AAV in the nucleus. Altogether, the results point toward a post-entry processing step that occurs suboptimal and therefore limits DC transduction.

### High-throughput selection of AAV peptide display library on iDC strongly selects for heparan sulfate proteoglycan-binding capsid variants

**Figure 2:**
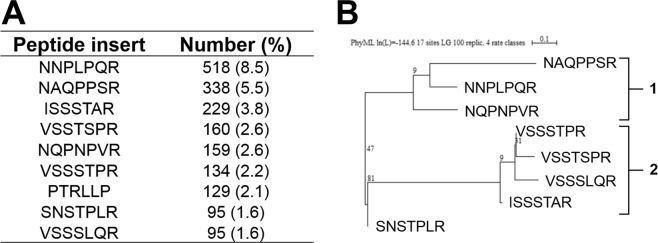
Selection of AAV capsid variants on iDC. (**A**) Quantification of NGS results. Variants that accumulated after two rounds of AAV2-based peptide library selection on iDC from different donors (Supplementary Fig. S3) were subjected to NGS using *cap*-specific primers to identify peptide sequences that mediated cell infection. Shown are sequences of peptide inserts present with amounts >1.5%. PTRLLP is excluded owning to its smaller size. (**B**) Phylogenic analysis of top AAV variants. Amino acid sequences of peptide inserts were aligned. A phylogenic tree was constructed using the maximum likelihood method. Selected variants were thereby classed into two main families. Numbers indicate bootstrap values.

Since the capsid is the main target of the intracellular processing of AAV particles, we performed an AAV peptide display library-based screening on iDC to select for better suited AAV capsid variants. The results of our serotype comparison (Fig. 1A) led us to choose AAV2 for the library backbone. Our library consists of capsid mutants displaying 7mer random peptides at position 587 of the *cap* ORF^23^. Peptide insertion at this position destroys the natural AAV2 heparan sulfate proteoglycan (HSPG) binding epitope, which is responsible for primary receptor binding^24–26^. In addition, we preselected our library by heparin affinity chromatography to enrich the library for variants displaying non-HSPG binding peptides^27^ and thus infect target cells through a novel, HSPG-independent pathway. Furthermore, we applied stringent selection conditions for our two selection rounds: (i) usage of iDC from different donors, (ii) short incubation time, and (iii) recovery of viral genomes from the nuclear compartment (Supplementary Figs S3A,B). DNA isolated following the nuclear fraction was analyzed by next generation sequencing (NGS) to identify and quantify the variants that accumulated in iDC (Fig. 2A).

Phylogenic analysis of the top variants, *i.e*. those that most strongly accumulated in the nuclear fraction of iDC, indicated that they could be classified into two families, “NNP” and “I/VSS”, with NNPLPQR as the strongest representative of the first, and VSSTSPR and ISSSTAR as lead candidates for the second (Fig. 2B). Of interest, although our library was pre-selected against HSPG-binding variants, all of the most frequently identified variants contained an arginine at peptide position 7, a characteristic feature of HSPG-binding capsids^22,26^. Based on this finding, we repeated the high-throughput screen on iDC, this time in the presence of heparin, a soluble analogue of HSPG. In addition, we reduced the incubation from 6 to 4 hrs (Supplementary Fig. S3B). Again, we observed a strong selection of variants displaying peptides belonging to the same two groups (Supplementary Fig. S3C). However, in the second screen, *i.e*. in the presence of heparin, in addition to members of the “NNP” or “I/VSS” families, motifs LPSRPSL and NSARPNS were also selected as top candidates. These peptides do not share the preference for “R” in position 7, but still possess an overall positive charge, a feature that in previous studies could be correlated with an HSPG binding phenotype^22,26^.

Since the two independent AAV peptide display screenings revealed a strong selection of variants belonging to the “NNP” or “I/VSS” family, we decided to focus on NNPLPQR, which was the most strongly selected motif in both screens, and ISSSTAR and VSSTSPR as prominent representatives of the “I/VSS” group. We produced the candidates as viral vectors encoding for GFP in a self-complementary genome conformation and named them according to the peptide insert (*e.g*. VSSTSPR: AAV with VSSTSPR insertion at position 587 of the *cap* ORF). All three variants were produced with yields and infectivity ratios comparable to the parental serotype (Supplementary Tables S1 and S2). In addition, we verified HSPG-dependency for cell transduction by a heparin competition assay on HeLa cells (Supplementary Fig. S3D).

### The maturation process of human DC strongly modulates AAV transduction efficiency

**Figure 3:**
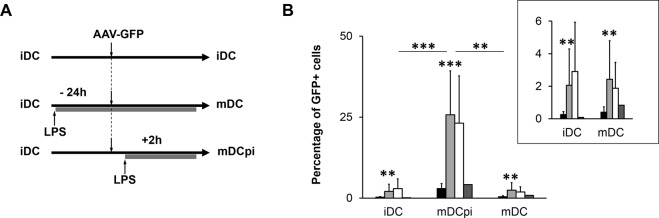
Influence of the DC maturation process on transduction. (**A**) Outline of the experimental procedure. DC maturation was induced by adding LPS (0.5–1 µg/ml) either 24 hrs before (mDC) or 2 hrs after (mDCpi) vector application. Cells were - in both cases - incubated for 24 hrs with AAV vectors. (**B**) Transduction efficiencies of AAV capsid variants. iDC, mDCpi or mDC were incubated with AAV2 (black bars), VSSTSPR (light grey bars), ISSSTAR (white bars) or NNPLPQR (dark grey bars) at a GOI of 10^4^ for 48 hrs. Cells were harvested and percentage of GFP^+^ cell was determined by flow cytometry. Inset: results obtained in iDC and mDC presented at increased scale. Error bars represent standard deviation of at least three independent experiments for AAV2, VSSTSPR and ISSSTAR. For NNPLPQR, two independent experiments were performed. Asterisks indicate the p value: *p < 0.05; **p < 0.01; ***p < 0.001.

We then focused on DC, our target cells, and compared our newly selected variants to the parental serotype. Since maturation of the capsid requires endosomal acidification and protease activity^2,3,22^ and since, in contrast to many other cell types, endosomal pH and proteolytic activity change with the state of DC maturation^28^, we tested iDC as well as more mature DC. Specifically, we incubated vectors with either iDC, or DC treated with lipopolysaccharide (LPS), a strong inducer of DC maturation, following two different protocols (Fig. 3A): either 24 hrs before incubation with the vectors (named here mDC for fully mature DC), or 2 hrs after vector addition (named here mDCpi for maturation post-infection). The effect of LPS on DC maturation was confirmed by immunofluorescence using high level of HLA-DR externalization as marker (Supplementary Fig. S4A).

Remarkably, the highest transduction efficiencies for all vectors were obtained with mDCpi and ranged between 25.7 ± 13.5% GFP^+^ DC for VSSTSPR and 2.9 ± 1.5% for AAV2 (Fig. 3B). This result strongly indicates that the maturation state influences DC permissivity to AAV transduction. Regardless of the DC maturation state, however, highest transduction efficiencies were obtained with VSSTSPR and ISSSTAR, the two variants belonging to the same group, and these were at least 10-fold more efficient than AAV2 (Fig. 3B, upper-right inset). We confirmed this improvement in transduction by fluorescence microscopy of vector-treated iDC using VSSTSPR as an example (Supplementary Fig. S4B). In contrast, the NNPLPQR variant that was selected with the highest frequency (and despite being highly infectious on HeLa cells, (Supplementary Fig. S3D and Table S2)) did not show significant improvement when compared to AAV2.

In order to exclude that differences in entry efficiency and/or intracellular distribution rather than the assumed endosomal maturation and intracellular processing were responsible for the enhanced transduction, we next compared distinct steps of the infection path of VSSTSPR, one of the two best performers, and of AAV2, as our reference, in iDC and mDCpi.

**Figure 4:**
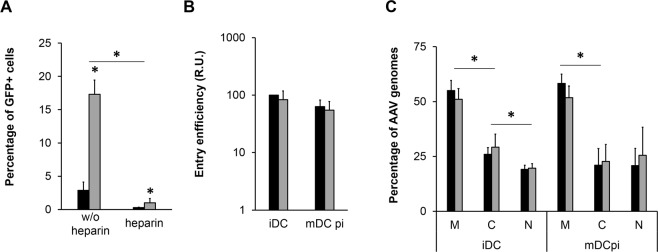
Comparison of AAV2 and VSSTSPR for DC transduction. (**A**) Heparin competition assay. mDCpi were incubated with AAV2 (black bars) or VSSTSPR (grey bars) at a GOI of 10^4^ in the absence or presence of heparin (25 µg per ml). Cells were harvested 48 hrs p.i. and the percentage of GFP^+^ cells was determined by flow cytometry. Error bars represent standard deviation (n = 4). (**B**) Entry efficiency of AAV vectors on iDC and mDCpi. DC were incubated with AAV2 (black bars) and VSSTSPR (grey bars) at a GOI of 10^4^ for 24 hrs. Cells were harvested by intensive treatment with trypsin and analyzed by qPCR using transgene-specific and β-globin-specific primers. Entry efficiency of AAV2 for iDC were set to 100%. Error bars represent standard deviation (n = 4). (**C**) Intracellular distribution of AAV in iDC and mDCpi. DC were incubated with AAV2 (black bars) or VSSTSPR (grey bars) delivering a single-stranded vector genome at a GOI of 10^3^ for 48 hrs. Cells were harvested by trypsin treatment and indicated cell fractions were isolated. Total DNA from each fraction was extracted and vector genome amounts were quantified by qPCR using transgene-specific primers. Error bars represent standard deviation (n = 3). Asterisks indicate the p value: *p < 0.05.

Firstly, we determined whether binding to HSPG is required for mDCpi transduction through a heparin competition experiment. As indicated in Fig. 4A, transduction of mDCpi with VSSTSPR and AAV2 was impaired in the presence of heparin, again confirming the importance of HSPG for DC transduction. Next, we compared immature and mature DC with regard to vector entry (Fig. 4B). For this, we incubated iDC and mDCpi with VSSTSPR and AAV2, respectively, at a GOI of 10^4^. As before, membrane-bound particles were removed, and vector genomes were quantified by qPCR. As indicated in Fig. 4B, iDC and mDCpi contained comparable amounts of vector genomes for both vectors, thus excluding preferential vector internalization as a possible reason for improved performance of both vectors in mDCpi compared to iDC, or of VSSTSPR compared to AAV2. Since DC maturation leads to profound structural reorganization of the endosomal compartment in addition to changes in the endosomal pH and activity of endosomal proteases^16,19^, we next compared the intracellular distribution of VSSTSPR and AAV2 in iDC and mDCpi (Fig. 4C). Again, we did not observe any differences, irrespective of the stage of DC maturation. However, the level of vector genomes in the nucleus tended to be higher in mDCpi than in iDC.

Since according to a recent report, DNA cross-contamination cannot be excluded when performing cell fractionation experiments^29^, we thoroughly re-evaluated our protocol. iDC were treated with AAV vectors or left untreated (mock) followed by cell fractionation. Fractions were analyzed in parallel by Western Blot (our standard procedure) and qPCR using genomic and mitochondrial DNA-specific primers. As indicated in Supplementary Fig. S5, a DNA cross contamination from the vesicular to the nuclear compartment was detectable in all samples. Thus, amounts of vector genomes in the nuclear compartment as shown in the side-by-side comparison of AAV2 and VSSTSPR are maybe overestimated. This, however, does not change our overall conclusion that the majority of AAV vector particles both, AAV2 and VSSTSPR, are retained in the vesicular compartment.

In summary, these results indicated that the maturation status of human DC strongly influences the transduction efficiency of AAV2 and of our selected capsid variants. For the latter, display of the I/VSS group peptides mediated significantly higher levels of transduction at all DC maturation stages tested here, arguing that these capsids have gained novel features.

### The I/VSS insertion affects capsid stability and improves vector uncoating in human DC

Since AAV2 and VSSTSPR were present in the nuclear fraction at comparable levels (Fig. 4C) but differed significantly with regard to DC transduction (Figs 3B and 4A), we hypothesized that they might differ in their uncoating efficiency, *i.e*. the efficiency of vector genome release from the capsid.

**Figure 5:**
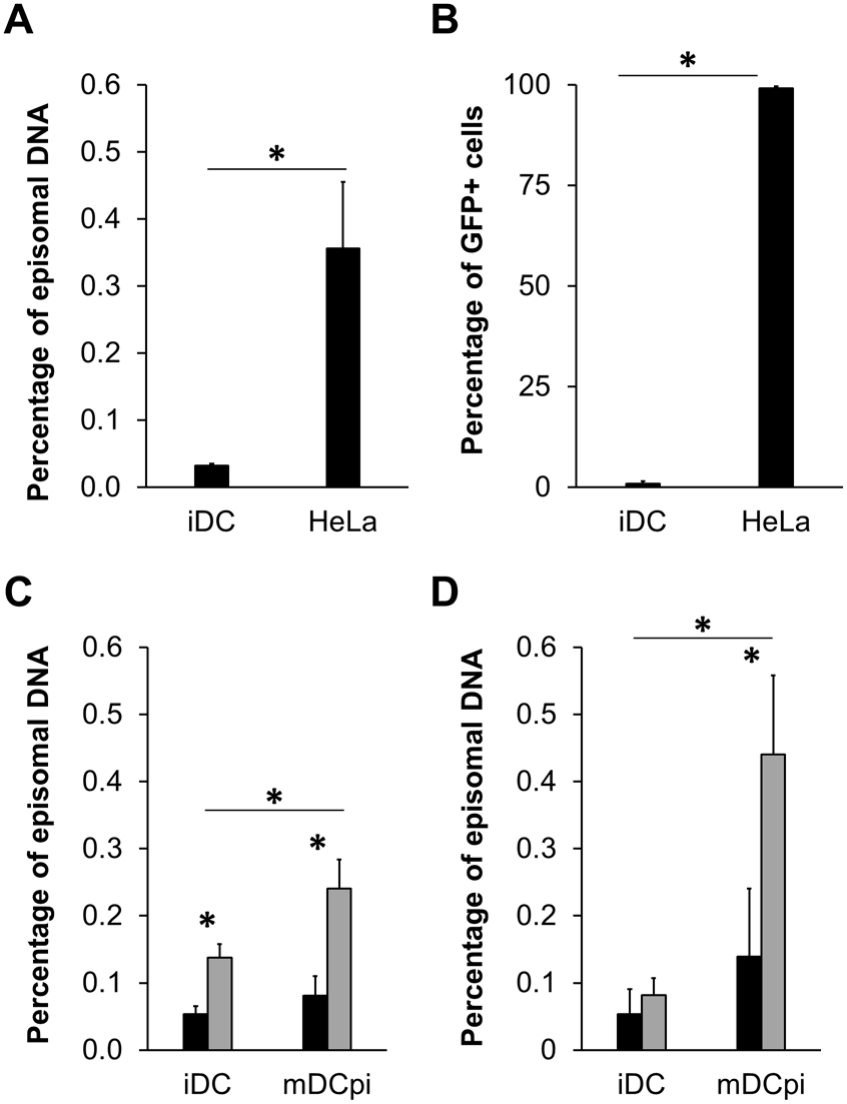
Quantification of episomal vector DNA as indication for uncoating efficiency. (**A**,**B)** Uncoating efficiency of AAV2. iDC or HeLa cells were incubated with AAV2 at a GOI of 10^4^ for 9 (**A**) or 48 hrs (**B**). Total DNA was extracted from cells harvested at 9 hrs p.i., DNA was then divided and either treated with T5 exonuclease or not, followed by quantification by qPCR. Cells harvested at 48 hrs p.i. were subjected to flow cytometry to determine the amount of GFP^+^ cells (Supplementary Fig. S6). (**C**,**D**) Uncoating efficiency of AAV2 and VSSTSPR on DC. iDC or mDCpi were incubated with AAV2 (black bars) or VSSTSPR (grey bars) at a GOI of 10^3^ for 24 (**C**) or 48 (**D**) hrs. Total DNA was extracted from cells harvested at 24 hrs p.i. and subjected to the uncoating efficiency assay as described in (**A**). Again, cells harvested at 48 hrs were analyzed by flow cytometry to determine the percentage of GFP^+^ cells (Supplementary Fig. S6). Error bars represent standard deviation (n > 3). Asterisks indicate the p value: *p < 0.05.

To evaluate this step, we developed an assay based on the ability of AAV genomes to form circular molecules (episomes) in the nucleus of the infected cell. The episomal form of the AAV DNA is very stable and cannot be degraded by exonucleases^30^. Therefore, the detection of AAV genomes following nuclease treatment can be used to indirectly measure the level of genome uncoating. For these analyses, we used the T5 exonuclease, which demonstrated a higher selectivity than plasmid-Safe^TM^ exonuclease for episomal DNA detection in related viral models^31^. Since HeLa cells are highly permissive for AAV2 and should therefore contain a detectable number of free vector genomes, these cells were chosen for comparison to iDC for development of this assay. Cells were first incubated with AAV2 at a GOI of 10^4^, followed by total DNA extraction at 9 hrs p.i. Half of the DNA was treated with T5 exonuclease, while the remaining half was left untreated. Subsequently, the amount of vector genomes was quantified by qPCR. At this time point, the percentage of AAV2 episomal DNA was at least 10-fold higher in HeLa cells compared to iDC (Fig. 5A), a result that correlates with respective transduction efficiencies (Fig. 5B). We then used this indirect assay to compare the uncoating efficiencies of VSSTSPR and AAV2 in iDC and mDCpi at 24 and 48 hrs p.i. The entry and the transduction efficiency were also monitored in parallel (Supplementary Fig. S6). These analyses indicated the presence of higher amounts of episomes in iDC and mDCpi transduced with VSSTSPR compared to AAV2 at 24 hrs p.i. (Fig. 5C). The difference in episomal DNA between VSSTSPR and AAV2 was also apparent at 48 hrs p.i. in mDCpi (Fig. 5D). Importantly, analysis of entry efficiencies again confirmed that these differences were not due to an increased level of vector uptake (Supplementary Fig. S6A,C). Furthermore, results correlated with the amount of GFP^+^ cells measured at both time points (Supplementary Fig. S6B,D).

The mechanism underlying the AAV uncoating process is still unknown. However, capsid stability is likely to impact uncoating efficiency^32,33^. Accordingly, our recent analysis of AAV physical properties by Atomic Force Microscopy (AFM) suggests that genome release can occur via two alternative pathways: either the capsid remains intact and ssDNA molecules are ejected, or the capsid is disassembled, leaving ssDNA in a compact entangled conformation^34^. To investigate whether VSSTSPR and AAV2 differ in this regard, we performed a destabilization assay.

**Figure 6:**
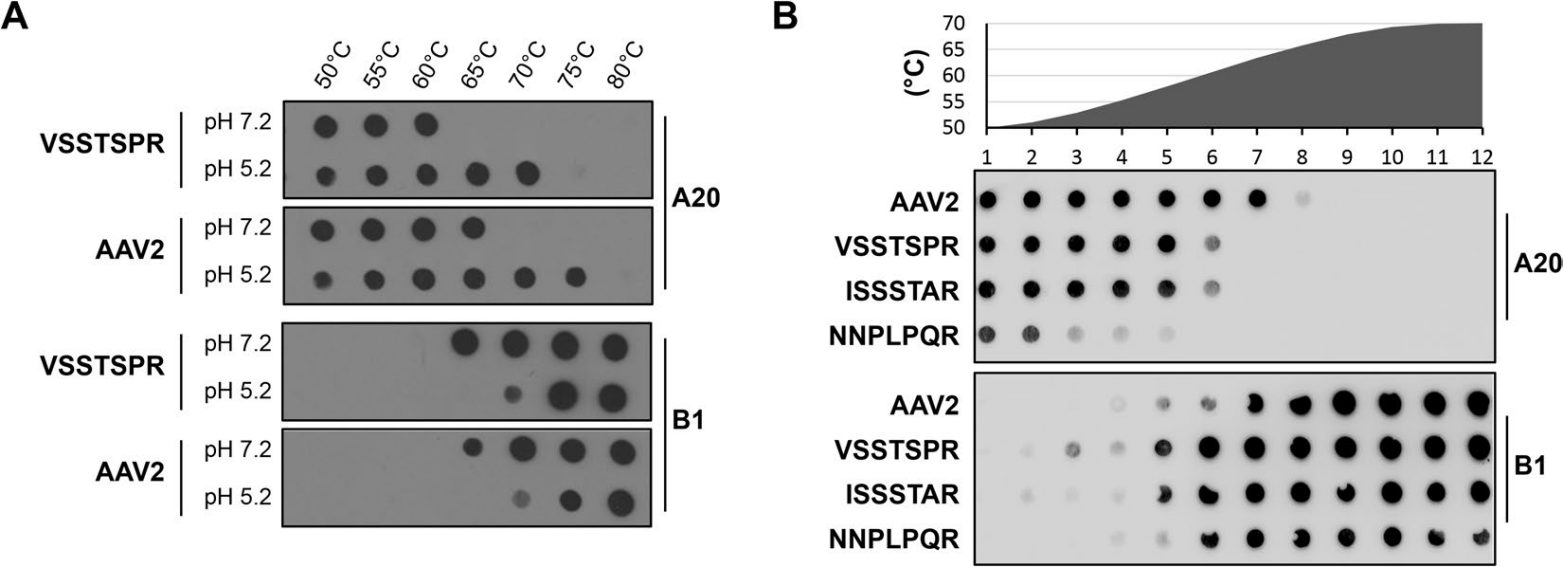
Thermal stability assay. (**A**) Capsid destabilization assay for AAV2 and VSSTSPR at different pH values. Vector preparations were adjusted in pH and then incubated for 15 minutes at indicated temperatures. A native dot blot was then performed using A20 and B1 antibodies to recognize intact and disassembled AAV capsids, respectively. Representative results for three independent experiments are shown (**B**). Comparative analysis of all capsid variants. AAV2, VSSTSPR, ISSSTAR and NNPLPQR were subjected to a temperature gradient followed by a native dot blot analysis. The integrity of the capsid was analyzed as described for A.

In brief, we subjected AAV particles to increasing temperatures at neutral (7.2) and acidic (5.2) pH followed by native dot blotting using A20 and B1 antibodies for detection of intact, *i.e*. assembled, and disassembled capsids, respectively (Fig. 6A). Interestingly, for both VSSTSPR and AAV2, capsids demonstrated a higher resistance to temperature in acidic compared to neutral conditions. Specifically, a significant number of the AAV2 particles were still intact at 75 °C and pH 5.2, while disassembled capsids were detectable at pH 7.2 already at 65 °C. The same trend was observed for VSSTSPR, however, at lower temperature. To determine whether the measured lower capsid stability is a unique feature for VSSTSPR, shared between the I/VSS family members, or a general property of capsids modified by peptide insertion at 587, we included ISSSTAR and NNPLPQR in a follow-up experiment and used the LightCycler ® apparatus to generate a temperature gradient to further increase the sensitivity of the assay (Fig. 6B). Again, the AAV2 capsid showed the highest resistance towards temperature, with most of the AAV2 particles still intact at 63 °C. In contrast, no detectable A20 signal was observed for VSSTSPR or ISSSTAR capsid variants at the same temperature. Based on the absence of detectable A20 and the strong B1 signals, it is conceivable to assume that capsids of the I/VSS group were already completely disassembled at this temperature, interestingly with similar A20/B1 patterns. In contrast, NNPLPQR showed a strikingly different pattern. Specifically, most of the NNPLPQR capsids lost the A20 signal at 51 °C, but were still not recognized by B1, a pattern that argues for a higher flexibility rather than complete denaturation of the capsid.

Altogether, these results indicated that insertion of peptides at position 587 affects the physical parameters of the capsid. In case of the I/VSS sequences, the capsid stability is reduced, which might favor genome uncoating and human DC transduction.

### Intra-muscular injection of VSSTSPR in mice improves transgene expression and increases immune responses

**Figure 7:**
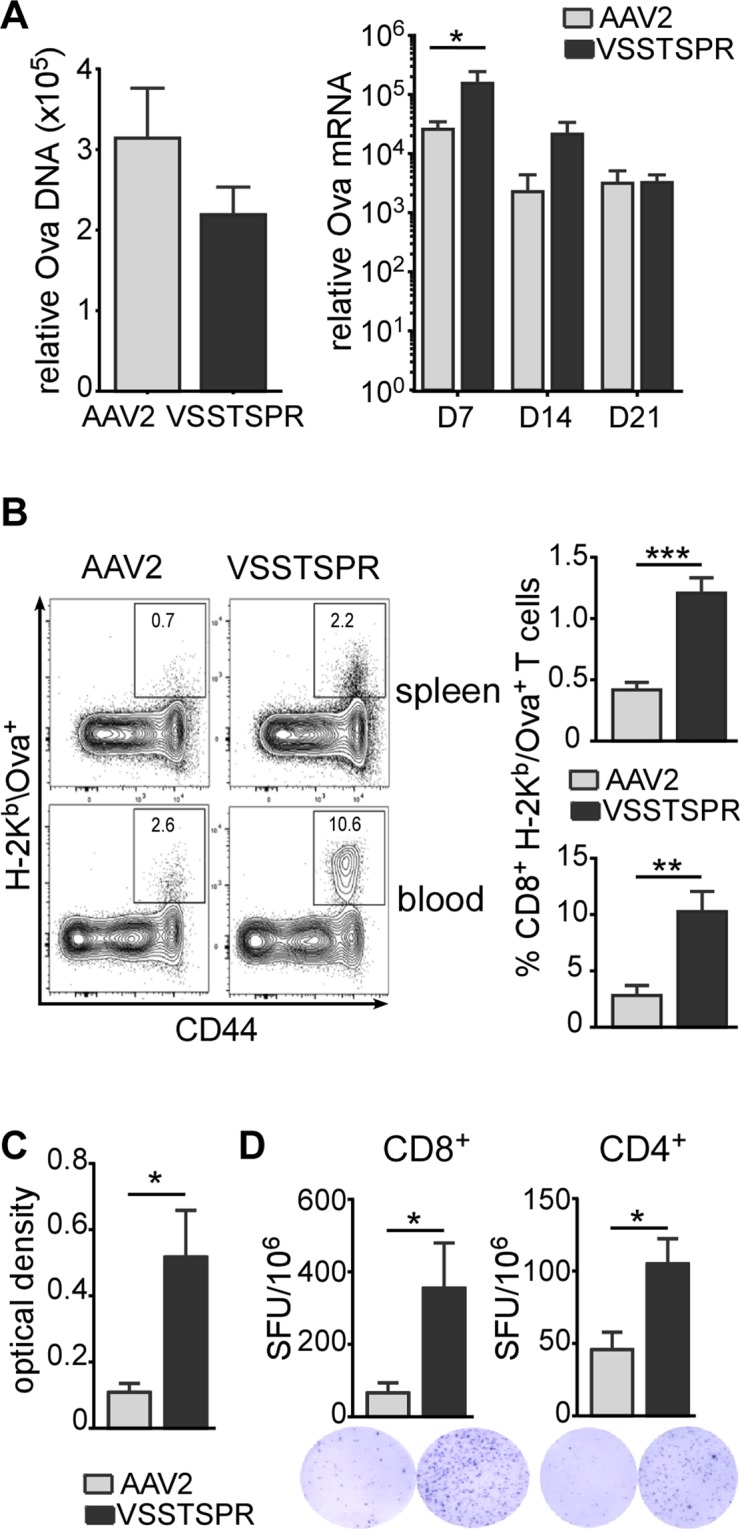
Characterization of transduction efficiency and immune responses following muscle gene transfer of AAV2-cOva and VSSTSPR-cOva. (**A**) Transduction efficiency. Gastrocnemius muscles were analyzed for the presence of vector genomes (at day 7; left panel) and corresponding mRNA expression (at days 7, 14 and 21, right panel) using Ova-specific primers. (**B**) Cellular immune responses. Representative Flow cytometry profiles and bar graphs (n = 5) showing the percentage of Ova-specific CD8^+^ T cells positively stained with H-2K^b^/Ova dextramers in spleen (upper panels) and blood (lower panels) 21 days after gene transfer. (**C**) Humoral responses. Sera were harvested from individual mice at day 21 and analyzed by ELISA to detect anti-Ova IgG. (**D**) Interferon-γ secreting cells. Bar graph and representative picture showing number of spot forming units (SFU) for 10^6^ splenocytes harvested at day 21 post gene transfer and re-stimulated with the immunodomiant MHC class I restricted Ova_257–264_ peptide (left panel) or MHC class II restricted Ova_323–339_ peptide (right panel) to stimulate interferon-γ secretion by antigen specific CD8^+^ and CD4^+^ T cells, respectively (n = 5). *p < 0.05; **p < 0.01; ***p < 0.001.

In order to investigate the impact of our capsid modifications on AAV performance *in vivo*, a comparative study between VSSTSPR and AAV2 was performed. For this, we used vectors encoding for the cytoplasmic form of ovalbumin (cOVA), an intracellular model antigen that offers the possibility to readily monitor AAV-induced immune responses^35,36^. We injected mice intra-muscularly with 3 × 10^9^ particles of VSSTSPR or AAV2 and quantified the level of transgene expression and of Ova-specific immune responses at day 7, 14 or 21 post gene transfer (Fig. 7).

Remarkably, quantification of transgene expression at day 7, which corresponds to an early time point when immune responses are still barely detectable, showed 6- to 10-fold higher transgene expression when VSSTSPR was used as the delivery tool (Fig. 7A, right panel). Quantification of the amount of transgene at the DNA level confirmed that similar doses of VSSTSPR vector particles, if not lower, had been injected (Fig. 7A, left panel), suggesting an improved  transduction efficiency.

We next evaluated the levels of immune responses induced upon intramuscular injection of these vectors. We first assessed the percentages of Ova-specific CD8^+^ T lymphocytes in blood and spleen 21 days post-vector delivery using flow cytometry (Fig. 7B). In the group injected with the VSSTSPR, we observed that a large fraction of CD8^+^ cells in the blood were directed against the Ova_257–264_ immunodominant peptide (10.3% in VSSTSPR group vs 2.8% in the control group, p = 0.0056, n = 5). Phenotypically, Ova-specific T cells were all contained in the fraction corresponding to activated or memory CD8^+^ cells expressing high levels of CD44 cell surface marker. For comparison, in the control group injected with AAV2, the percentages of anti-Ova CD8^+^ T cells in spleen and blood compartments were at least 3-fold lower. We also monitored the humoral immune responses in these mice by measuring the presence of anti-Ova IgG by ELISA. Results confirmed significant increased levels of anti-Ova IgG in the sera of mice injected with VSSTSPR (Fig. 7C). As production of IgG requires activation of CD4^+^ helper T cells, this suggested that anti-Ova CD4^+^ T cells were also increased in this group. To directly evaluate this possibility, we estimated the levels of splenic T cells responding to MHC class I and MHC class II restricted immunodominant Ova peptides using ELISpot assays. In line, significant higher numbers of T cells from the VSSTSPR group secreted IFNγ when restimulated *in vitro* with either one of these peptides (Fig. 7D). Thus, higher levels of CD4^+^ and CD8^+^ immune responses were induced in the group injected with the VSSTSPR capsid variant. These findings were consistent with the progressive decline of *Ova* mRNA observed between days 7 and 21 post gene transfer (Fig. 7A, right panel), most probably reflecting elimination of transduced muscle cells by infiltrating functional cytotoxic CD8^+^ immune cells.

Next, we focused on the vector itself and investigated whether VSSTSPR induces anti-capsid cytotoxic CD8^+^ T cells. For that, we injected VSSTSPR and AAV2, respectively, intramuscularly, and assessed the capsid-specific CD8^+^ T lymphocytes responses by an ELISPot assay using the immunodominant common capsid-derived PL8 peptide. VSSTSPR induced a significant higher anti-capsid cellular response compared to AAV2 as clearly indicated by the higher number of IFNγ secreting T cells responding to the PL8 peptide *in vitro* (Supplementary Fig. S7).

Altogether, these results indicate that VSSTSPR transduces cells with higher efficiency and that novel properties of the VSSTSPR variant correlate with a higher level of antigen-specific antibody and cellular immune responses directed against the transgene product and against the capsid.

## Discussion

As for other viral vectors, AAV’s interaction with DC constitutes the critical parameter that controls long-term transgene expression^20^. As DC were previously described as refractory toward efficient productive AAV vector transduction, we aimed to identify barrier/s that limit this process. Our results indicate that vector uncoating is severely impaired in human DC. We also show that DC maturation greatly improves efficacy of vector transduction, in particular during its intermediate phase, suggesting that intracellular processing of AAV vectors in immature DC is suboptimal. A high-throughput selection screen of our AAV2-based capsid library identified variants that overcome such barriers through display of distinct peptide motifs and a subsequent change in the vector-DC interaction. Finally, we show that these modifications of the viral capsid impact the level of adaptive immune responses induced *in vivo*.

DC are professional APC. They differentiate from bone-marrow progenitor cells into either plasmacytoid (pDC) or pre-conventional DC (pre-cDC)^37^. The latter migrate via the blood stream into lymphoid and non-lymphoid tissues, where they differentiate further into different subsets of cDC^38^. Here, we focused on human iDC, a subset of DC generated *in vitro* by culturing blood monocytes in the presence GM-CSF and IL-4. iDC are considered as a suitable model system to study human DC biology and function^39^. Like *in vivo* derived immature DC, iDC endocytose exogenous particles^40^. Well-described receptors in this regard are HSPG, the primary or attachment receptor for AAV2, expressed on iDC in the form of glypican-1, -4, -5, and syndecan-1, -4^41^, and α_v_β_3_ and α_v_β_5_ integrin^40,42^. The latter represents one of AAV2’s internalization receptors^42^ that is engaged by the viral capsid after binding to HSPG^43^, likely explaining why this serotype accumulated in iDC with higher efficiency than other AAV serotypes (Fig. 1A). The HSPG motif is unique for AAV2 and located at the 3-fold symmetry axis. Residues R448/585 and R451/588 from one capsid subunit and R350/487 from another are critical for binding the negatively charged sugar hydroxyls of HSPGs^24,25,44^. This motif is destroyed when inserting peptide ligands at N587^26,45^. In addition, variants that re-gain the ability to bind to HSPG via the peptide insertion can be counter-selected from the library by heparin affinity chromatography as accomplished in this study^27^. Interestingly, despite these two measures, capsid variants were selected that involved HSPG for cell infection as demonstrated by the heparin competition assay performed on HeLa and on DC (Supplementary Fig. S3D and Fig. 4A). Furthermore, repeating the screening in the presence of heparin to further increase the selection pressure for non-HSPG-binding variants resulted in selection of the same capsid variants, revealing the importance of this surface molecule for the AAV-iDC interaction (Supplementary Fig. S3C). Based on this, we may speculate that HSPG binding is required for guiding our capsid variants into an intracellular pathway that leads to nuclear accumulation, a feature we specifically selected for since we isolated viral genomes from the nuclear fraction of iDC (Supplementary Fig. S3A). Indeed, HSPG-mediated transport of various cargos, including chemokines, cytokines, growth factors as well as viral proteins, to the nucleus has been described^46,47^. An alternative explanation could involve structural rearrangement of the capsid induced upon HSPG binding, which revealed modifications at the protrusions and 5-fold axes of symmetry^43^. The latter regions contain the pores that are widened as a consequence of HSPG binding and thus may prime the viral capsid for uncoating^43^.

Besides adjustments in capsid structure induced at the cell surface, capsid binding to receptors initiates particle uptake through clathrin-dependent endocytosis as well as rearrangements of the cytoskeleton that prepare for the transport of AAV vector-containing vesicles^43,48^. Clathrin-mediated endocytosis and the CLIC/GEEC clathrin-independent endocytic pathway are the two main entry routes described for AAV^22,49,50^. Once inside an endosome, AAV particles are transported within the vesicular system towards the trans-Golgi network (TGN). This involves trafficking from early to late and/or recycling endosomes before TGN is reached^50^. On this route, a second conformational change of the capsid – induced by a low endosomal pH and proteases - further prepares the capsid for nuclear entry and uncoating^2,3,51–53^. Given the importance of the intracellular vesicular system, it is not surprising that the majority of intracellular AAV particles are found within the membrane fractions of both HeLa and DC (Figs 1 and 4). Interestingly, the intracellular distributions of AAV2 and VSSTSPR in DC did not differ significantly despite a one-log higher transduction efficiency of the latter. This argues that our capsid variant follows the same intracellular pathway with similar efficiency (at least based on the resolution offered by bio-distribution assays). The higher transduction efficiency of the VSSTSPR variant (Fig. 3B) correlated instead with a qualitative and quantitative difference in vector stability and vector uncoating (Fig. 5C,D). This feature seems to be shared by the members of the I/VSS family, which showed a similar capsid stability in our thermostability assay (Fig. 6) and a comparably higher transduction efficiency on DC (Fig. 3B).

Under normal steady-state conditions, DC exist in an immature form particularly efficient to capture and uptake exogenous particles and antigens. Therefore, DC are endowed with a continuous endocytic activity and are equipped with a specialized antigen processing machinery. Specifically, in order to avoid complete degradation of endocytosed particles and/or antigens, the pH in phagosomes and endosomes is less acidic in DC than in other phagocytic immune cells. Also, the pH remains alkaline (pH 7 to 7.5) during the first three hours after particle uptake in part by recruiting the NAPDH oxidase NOX2 and inactivating V-ATPase (the main proton transporter in lysosomes). As a consequence, most proteases are less efficient in these sub-optimal pH conditions^16,54^. In light of the dependency for an acidic pH and proteases, it is conceivable that the endosomal compartment in iDC is not optimal to prepare the capsid for uncoating. In this scenario, only a very minute population of AAV2 particles reaching the nuclear compartment would be capable to uncoat and to contribute to transduction. Insertion of peptides of the I/VSS family, however, may have changed the capsid properties and favor vector uncoating despite the suboptimal endosomal conditions encountered in iDC. Alternatively or maybe additionally, use of I/VSS binding receptor (instead of natural receptors for AAV) for cell entry induces an intracellular signal pathway that promotes AAV endosomal processing. This latter assertion is based on the finding that DC process antigens differentially depending on the specific receptor engaged upon pathogen encounter^16^.

Interestingly, AAV transduction efficiency was further enhanced through LPS-mediated stimulation of iDC 2 hrs p.i. (Fig. 3B). Enhancement was similarly efficient for both I/VSS engineered vectors, NNPLPQR, and AAV2, hinting towards a general enhancement of AAV processing. Stimulation of iDC by LPS or other agents that induce their maturation, is known to be accompanied by a profound reorganization of the endosomal compartment, including augmented endocytosis and phagocytosis during its early phases, as well as an enhancement in antigen translocation into the cytosol followed by changes in endosomal pH and protease activities^16,54,55^. These changes may result in an endosomal environment that is more appropriate albeit still not optimal for AAV particle processing, and might explain why iDC stimulation 2 hrs after AAV transduction strongly increased transduction efficiency independently of the capsid (Fig. 3B). Whether it is a matter of pH, availability of proteases or modification of intracellular pathways used by the vector, remains to be investigated. Of note, infection of fully mature DC resulted in lower transduction levels than those measured in iDC (Fig. 3B). This most probably reflects the known lower capacity of fully mature DC to uptake and internalize antigens and particles (data not shown) as well as a possible modification in the intracellular environment^16^.

As transduction of DC can have an important impact on immune responses, we next evaluated this aspect using cOva as transgene^35,36^. Our results indicate that cOva delivered by VSSTSPR resulted in a robust immune response consisting of antibody and CD8^+^ T-cell responses against the transgene product (Fig. 7C,D). Regarding the anti-capsid cellular immune response, our results demonstrated that VSSTSPR induce a more prominent anti-capsid CD8^+^ T cell response as compared to AAV2 (Supplementary Fig. S7). Whatever the precise mechanism is, this may in turn increase the immune responses against the encoded transgenic protein. Taken together, these features may qualify capsids displaying peptides of the I/VSS family as scaffolds for the design of novel vaccines.

Interestingly, we observed that at early time points (day 7), transgene expression occurred at a higher level with VSSTSPR compared to AAV2 (Fig. 7A, right panel). Although this remains to be investigated, the improved transduction efficiency of VSSTSPR may have resulted in antigen presentation by transduced muscle cells, which are known to express MHC class I and class II molecules in some circumstances and can thus behave as surrogate APC.  In addtion, transduction of muscle-resident DC and/or DC localized in the draining lymph nodes may have also contributed to the higher immune responses^56,57^. However, even if we have chosen a cytoplasmic form of Ova, we cannot completely exclude that the higher level of expression observed with the VSSTSPR variant caused a higher level of cOva release by dying muscle cells and thereby improved presentation and/or cross-presentation by the surrounding APC to T cells^12,58^.

In summary, our study reveals for the first time that genetic engineering can be used to improve capsid uncoating, which we demonstrate is a major barrier that limits AAV-mediated transduction of DC and possibly of further cell types. Additionally, this work shows the importance of improving our knowledge about factors that govern capsid stability and vector uncoating as these appear to have considerable impact on the efficiency of AAV-mediated gene therapy and vaccination approaches.

## Material and Methods

### Ethics statement

Human blood was purchased from “Etablissement Français du sang” (EFS). Monocytes were derived from these samples and were differentiated as outlined below. All animal studies were conducted in accordance with the guidelines of local institutional care and ethical committee and following the national and European directives. Animal protocols were approved by the committee for research and ethics and the French ministry (N/02-02-11/06/02-511 14 and 01979.03).

### Cell lines and primary cells

The human cervix carcinoma cell line HeLa (kindly provided by Alessandra Recchia, UNIMORE, Modena) and human embryonic kidney cell line HEK-293 (kindly provided by Philippe Moullier, INSERM U1089, Nantes) cells were maintained in Dulbecco’s modified Eagle’s medium (DMEM; Life Technologies) supplemented with 10% fetal bovine serum (FCS; HyClone) and 1% penicillin-streptomycin (P/S; 5,000 U/ml; Invitrogen), and cultivated at 37 °C, 5% of CO_2_. Monocytes were purified from peripheral blood (purchased from EFS) of healthy donors by two successive density gradients (Ficoll (GE Healthcare Life Sciences) and Percoll (GE Healthcare Life Sciences). Differentiation of monocytes into iDC was performed by incubating monocytes for 5 days in DC medium (RPMI with, 10% of FCS, 1% P/S, 10 mM HEPES, 2.5 mM NEAA; 1 mM sodium-pyruvate and 0.05 mM β–mercaptoethanol) complemented with IL-4 and GM-CSF (100 ng/ml). The differentiation status of iDC was verified by FACS analysis, using the level of CD209 (DC-Sign, MACS), CD14 (BD Biosciences) and CD86 (BD Biosciences) as markers. Every experiment was performed with iDC derived from at least two different donors and two AAV vector batches.

### *In vitro* AAV peptide display selection on DC

AAV capsid variants were selected by screening an AAV2 peptide display library^23^ on iDC. Library was subjected to a phenotype/genotype coupling step followed by heparin affinity chromatography^27^. Flow-through was concentrated and again purified by iodixanol density gradient centrifugation. This pre-selected library was used for two independent screens each consisting of two rounds of selection. In the first screen, cells were transduced with the library at GOI 10^3^ and harvested 6 hrs p.i. Cells are then fractionated as described below and AAV viral genomes that accumulated in the nuclear fraction were used to generate a new library. For this, viral DNA was amplified using primers flanking the peptide insertion site allowing to re-clone the target sequences into the pLG shuttle plasmid (Zhang *et al*., manuscript in preparation). Sublibrary was produced as described above and screened on iDC from a different healthy blood donor. After cell fractionation, viral DNA from the nuclear fraction was sequenced by NGS on the 454-pyrosequencing platform (GS Junior, Roche). For the second screening, heparin was added during selection and time to harvest was reduced to 4 hrs p.i. (Supplementary Fig. S3). Based on the NGS results, candidates were picked as described and respective peptide sequences were introduced into the *cap* ORF of pRC’99^59^.

### AAV vector production

Stocks of recombinant AAV2 particles were generated by calcium phosphate transfection of HEK-293 cells followed by gradient purification^60,61^. The number of vector/viral genome containing particles per milliliter (vg/ml) was determined by qPCR using transgene or *cap* ORF specific primers, while the transducing titer was determined by serial dilution of viral vectors assayed on HeLa cells followed by flow cytometry analysis as described^27^. Genomic titers of various vector preparations are presented in Supplementary Table S1. If not indicated otherwise, all experiments were performed with AAV vectors delivering a vector genome in the self-complementary conformation.

### Cell transduction assays

Cells were plated and then transduced with vector particles diluted at the indicated GOI (vg/cell) in complete medium. At the indicated time point, cells were harvested by extensive trypsin treatment and washed in PBS^22^. When indicated, iDC were additionally treated with LPS (0.5–1 µg/ml). For competition assays (Fig. 4), the medium containing vector particles was supplemented with heparin (25 µg/ml). Subcellular fractionation was performed using Subcellular Protein Fractionation Kit for Tissue (ThermoFisher Scientific). Purity of fractions on the protein level was confirmed by Western blot (Supplementary Fig. S2) using anti-Rab 5 (Santa Cruz sc 46692; 1:100), anti-Tubulin (SIGMA T5198; 1:5000), anti-Lamin B1 (Abcam antibody 16048; 1:5000), and anti-Calreticulin (Affinity BioReagents PA3-900, 1:100) antibodies, respectively. Factions were spiked with 1 ng of murine TOPO-GAPDH plasmid followed by DNA extraction (Blood & Tissue kit, Qiagen). The qPCR reactions were conducted with the FastStart universal SYBR green master reagent (Roche Diagnostics) on the Step One Plus real-time PCR system (Applied Biosystems) or LightCycler® 96 System (Roche Diagnostics). All samples were run in duplicate, and the results were analyzed using ABI StepOne software v2.3 or LightCycler® 96 System software 1.01.01.0050 (Roche Diagnostics). Primers used to quantify viral or vector genomes or for normalization are listed in Table 1.

**Table 1:** List of primers.

Primers	Sequence
GFP-R	ACG ACG GCA ACT ACA AGA CC
GFP-F	CTC CTT GAA GTC GAT GCC CT
β-Globin-F	CCC TTG GAC CCA GAG GTT CT
β-Globin-R	CGA GCA CTT TCT TGC CAT GA
mGAPDH-F	GCA TGG CTT TCC GTG TTC
mGAPDH-R	TGT CAT CAT ACT TGG CAG GTT TCT
OVA-F	AAG CAG GCA GAG AGG TGG TA
OVA-R	GAA TGG ATG GTC AG CCC TAA
PLAT-F	ACC TAG ACT GGA TTC GTG
PLAT-R	AGA GGC TAG TGT GCA T

For immunofluorescence analyses, iDC were harvested and plated in a 96-Well Optical-Bottom Plates (Fisher Scientific) or in a Labtek, both pre-coated with poly-lysine, followed by antibody staining using standard protocols^62^. Antibody used were: A20^63^, (1:50), anti-HLA-DR (anti human HLA DR-biotin clone L243, Novus Biologicals) used at 1/1000 dilution, and anti-Lamin B1 (Abcam antibody 16048; 1:5000). Nuclei were stained with DAPI or Hoechst as specified in the figure legend.

### *In vitro* uncoating assay

Briefly, cells treated with AAV vectors were harvested at the indicated time points, washed with PBS, extensively treated with trypsin and washed again. Then the DNA was extracted (Qiagen kit) and eluted in 100 µL. Twenty-five µL of DNA were treated with T5 exonuclease (Biolabs, M0363, 30 Unit) at 37 °C overnight, then incubated 10 min at 70 °C and diluted 2 times to inactivate the enzyme. In parallel, 25 µL of the same DNA sample were mock-treated. The quantification of vector genomes was performed by qPCR using transgene (GFP)-specific primers. The percentage of episomal DNA was calculated from the ratio of T5 resistant GFP DNA to total GFP DNA.

### Capsid thermal-stability assay

For the first assay (Fig. 6A), 10^8^ vg of indicated AAV vectors were adjusted to the “pH buffer” (citric acid/Na_2_HPO_4_) at pH 7.2 and pH 5.2, for 15 min. at room temperature. Then samples were exposed to indicated temperatures (50, 55, 60, 65, 70, 75, 80 °C) for 15 min and then diluted in PBS. For the second assay (Fig. 6B), wells of a qPCR plate were loaded with 5 × 10^8^ vg of indicated AAV vectors diluted in PBS. The temperature gradient was generated by a LightCycler® 96 System (Roche Life Science) using a self-designed program (Supplementary Table S3). After the run, PBS was used to dilute samples. In both experiments, after PBS dilution, samples are transferred to a nitrocellulose membrane using a vacuum blotter for a native dot blot assay. After saturation, the membranes were incubated overnight at 4 °C with A20, or B1 antibodies^63^. A horseradish peroxidase-conjugated anti-mouse antibody (Sigma,1/10,000 dilution) was then applied for 1 h at room temperature. Finally, the membranes were incubated with an enhanced chemiluminescence reagent (West Dura; Pierce) and analyzed by autoradiography film exposure or FusionFX device (Peqlab).

### Animal experiments and analyses

Female C57BL/6 mice were obtained from Janvier Labs (Le Genest Saint Isle, France). Mice were all between 8–10 weeks of age at the beginning of the experiments and were housed in our animal facility in a specific pathogen-free barrier facility. The following fluorochrome-conjugated monoclonal antibodies were used for phenotypic analysis of mouse T cells by flow cytometry: FITC anti-CD44 (IM7), PerCP/Cy5.5 anti-CD45 (30-f11), APC anti-CD8α (53–6.7), APC-Cy7 anti-CD4 (G41.5) (all from Sony biotechnology). PE-conjugated H-2K^b^/Ova_257–264_ dextramers were used to detect CD8^+^ T cells that specifically recognize the immunodominant Ova_257–264_ peptide (Immudex, Copenhagen, Denmark). Single-cell suspensions derived from spleen, lymph nodes, or peripheral blood were analyzed by flow cytometry using a FACSCanto-I or an LSRFortessa (BD Biosciences), and using FlowJo software (Tree Star, Ashland). Anti-Ova IgG antibodies were detected by ELISA as previously described^35,36^. ELISpot were used to quantify the numbers of Ova-specific CD8^+^ or CD4^+^ T cells or anti-capsid-specific CD8^+^ T cells secreting IFNγ upon *in vitro* re-stimulation as previously described^35,36^. Briefly, 10^5^ to 2.5 × 10^5^ splenocytes per well were cultured overnight in RPMI medium in the presence of 10 μg/ml Ova_257–264_ or Ova_323–339_ peptides for detection of MHC class I or MHC class II restricted CD8^+^ or CD4^+^ T cells Ova-specific T cells, respectively, or immunodominant PL8 peptide (PQYGYLTL). Cultures were stopped 16 to 20 hrs later and treated according to manufacturer’s instructions (Diaclone, Besançon, France). Number of spots in each well was analyzed with an Enzyme-linked immunospot plate reader and a dedicated ImmunoSpots software (C.T.L., Bonn, Germany). Ova DNA and corresponding Ova mRNA were quantified from transduced muscles by qPCR using SYBR green Mastermix (Roche, Meylan, France)^35,36^. Values were normalization using eukaryotic translation elongation factor 2 (Eef2) as target. All qPCRs were performed using a LightCycler 480 apparatus (Roche Diagnostics).

### Statistical analysis

Non-parametric test was performed for statistical comparison between groups using one-way analysis of variance (Kruskal-Wallis test), this test was performed on Stata (vs13) software. P values are indicated as follows *p < 0.05; **p < 0.01; ***p < 0.001.

## Supplementary information

Supporting information

## Data Availability

The datasets generated during and/or analyzed during the current study are available from the corresponding author on reasonable request.
