# Supplementary material for: Vector uncoating limits adeno-associated viral vector-mediated transduction of human dendritic cells and vector immunogenicity
Source: Sci Rep. 2019 Mar 6;9:3631. doi: 10.1038/s41598-019-40071-1 (PMC6403382; doi:10.1038/s41598-019-40071-1)
Supplement: Supplementary file 1 — Supporting information [file 41598_2019_40071_MOESM1_ESM.docx]

Supplementary files

Title: Vector uncoating limits adeno-associated viral vector-mediated transduction of human dendritic cells and vector immunogenicity

Axel Rossi, Léa Dupaty, Ludovic Aillot, Liang Zhang, Célia Gallien, Michael Hallek, Margarete Odenthal, Sahil Adriouch, Anna Salvetti, Hildegard Büning


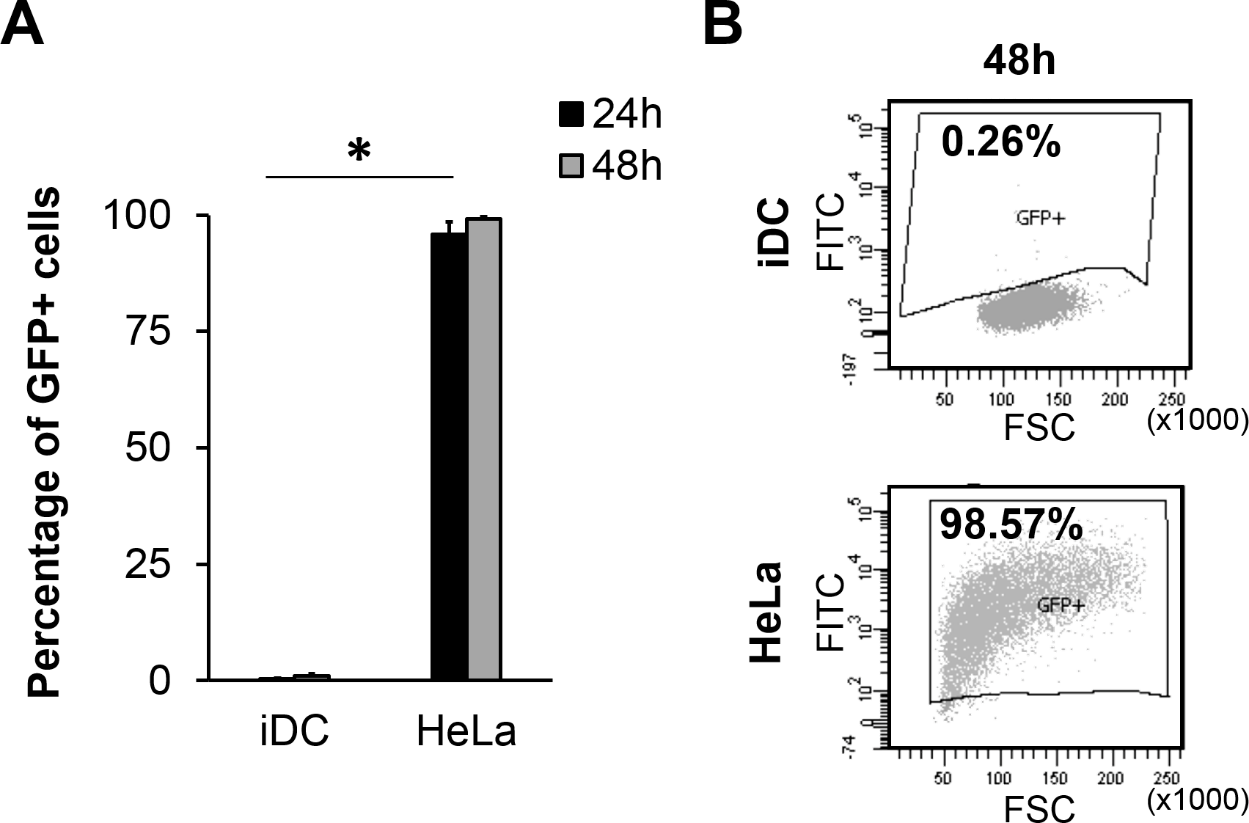


**Supplementary Figure S1. Transduction assay on iDC. (A)** Transduction efficiency of AAV2. iDC or HeLa cells were incubated with AAV2 vectors encoding for enhanced green fluorescent protein (GFP) at a GOI of 10^4^ and analyzed by flow cytometry at 24 and 48 hrs p.i., respectively. **(B)** Representative example of raw data of flow cytometric analysis. Threshold was set according to non-treated control cells. Error bars represent standard deviation (n=3), asterisks indicate the p value: [*****]: p<0.05.

**Supplementary Figure S2. Intracellular distribution of AAV2 in iDC. (A)** Western blot analysis of subcellular fractions from iDC incubated with AAV2 vectors. Purity of fractions was confirmed using antibodies directed against key proteins: Tubulin for the cytosolic fraction, Calreticulin and Rab5 for membrane fraction and Lamin B1 for the nuclear fraction. Shown are results of one representative analysis. M, membrane; C, cytosol; N, nucleus; T, total extract. **(B)** Quantification of AAV vector genomes in subcellular fractions from iDC. AAV2 encoding for GFP were incubated with iDC at a GOI of 10^3^. Twenty-four hrs p.i., cells were washed, harvested, and indicated cellular compartments were isolated. Vector genomes contained in each compartment were quantified by qPCR. Data represent the mean of two independent experiments. **(C)** Quantification of AAV vector genomes in subcellular fractions from iDC. AAV2 encoding for GFP in a single-stranded vector genome conformation were incubated with iDC at a GOI of 10^3^. Forty-eight hrs p.i., cells were washed, harvested and indicated cellular compartments were isolated. Vector genomes contained in each compartment were quantified by qPCR. Data represent the mean of two independent experiments.

**Supplementary Figure S3. Selection of AAV capsid variants. (A)** Overview on the selection procedure. **(B)** Details on experimental conditions for the two independent AAV peptide display selections. To avoid a donor-related bias, for each round iDC from a different donor was used. To increase the selection pressure, the second high-throughput selection screening was performed in the presence of heparin (hep). In addition, the incubation time was reduced from 6 to 4 hrs. **(C)** Result of NGS analyses of second screening. Bold R mark arginine residue of 7mer peptide insert contributing to the HSPG binding RXXR motif that is formed at position 587 of our library (for details see ^1^). **(D)** Heparin competition assay on HeLa cells. HeLa cells were incubated with indicated vectors at a GOI of 10^4^ with or without heparin (425 I.U.). Cells were harvested 48 hrs p.i. and analyzed by flow cytometry (n=1).

**Supplementary Figure S4. Microscopic analyses of DC. (A)** Immunofluorescence analysis of immature and mature DC. iDC were left untreated or were incubated with LPS according to the protocol depicted in Fig. 3. Cells were fixed, permeabilized, and stained with anti-HLA-DR and anti-Lamin B1 antibodies, respectively. After incubation with labeled secondary antibodies, the cells were imaged by confocal microscopy. The nuclei were labeled with Hoechst **(B)** VSSTSPR and DC. iDC and mDCpi were incubated with VSSTSPR at a GOI of 10^4^ for 48 hrs. Cells were analyzed by light and fluorescence microscopy.

**Supplementary Figure S5. Analyses on possible DNA cross-contamination.** Francois and colleagues reported on limitations of current cell fractionation protocols ^2^. We therefore analyzed iDC (n=5, labelled 1 to 5) treated with AAV vectors or not, and subjected to our cell fractionation protocol. **(A)** Western blot analysis of subcellular fractions from iDC. Quality of fractionation on the protein level was analyzed using antibodies directed against marker proteins: Tubulin (“Tub”) for the cytosolic fraction, Rab5 for membrane fraction and Lamin B1 for the nuclear fraction. **(B)** Quantification of genomic and mitochondrial DNA in subcellular fractions. qPCR was performed using Plasminogen Activator (PLAT)-specific (Table 1) and mitochondrial DNA (“Mito”)-specific primers ^3^. Shown are the raw data. “C” = cytosolic, “M” = membrane and “N”= nuclear fraction. Numbers indicate the iDC sample. **(C)** Quantification of genomic and mitochondrial DNA in subcellular fractions. Raw data from (**B**) presented as mean values and normalized to nuclear faction. Error bars represent standard deviation (n=5).

**Supplementary Figure S6. Transduction and entry efficiencies on DC.** Indicated vectors were incubated with iDC and mDCpi at a GOI of 10^3^. Cells were harvested 24 (**A** and **B**) or 48 (**C** and **D**) hrs p.i. AAV entry efficiency (**A**; **C**) was measured by qPCR on total DNA using transgene-specific and β-globin-specific primers. The percentage of GFP^+^ cells was determined by flow cytometry (**B**; **D**). Error bars represent standard deviation (n > 3). Asterisks indicate the p value: [*****]: p<0.05; [******]; p< 0.01.





**Supplementary Figure S7. Characterization the anti-capsid immune response following muscle gene transfer of AAV2-cOva and VSSTSPR-cOva**.

Mice (n=5/group) were injected with 3x10^9^ vg/mouse of either AAV2-cOva or VSSTSPR-cOva in the gastrocnemius muscles. Animals were killed either 14 days (left panel) or 40 days (right panel) later to harvest the spleen and to evaluate the cellular immune response directed against the AAV capsid proteins by ELISpot assays. For that, 2.5 × 10^5^ splenocytes per well were re-stimulated *in vitro* in presence of 10 µg of the immunodominant PL8 peptide (PQYGYLTL) and cultured overnight before accessing the number of interferon-γ secreting cells. Bar graphs represent the mean numbers of spot forming units (SFU) interferon-γ secreting cells for 10^6^ splenocytes obtained at day 14 (left panel) or at day 40 (right panel) post gene transfer. Representative ELISpots pictures are also shown under the corresponding bar graphs. [**]: p< 0.01.

**Supplementary Table S1. Genomic titers of indicated AAV vector preparations**

| **Vector** | **Genome**  **conformation** | **Genomic titers**  **(vg/ml)** | **Number of**  **preparations** |
| --- | --- | --- | --- |
| **AAV2** | sc | 6.84+2.55 x10^10^ | 5 |
|  | ss | 8.29+2.85 x10^10^ | 2 |
| **VSSTSPR** | sc | 2.58+2.07 x10^11^ | 5 |
|  | ss | 9.36+5.15 x10^10^ | 2 |
| **ISSSTAR** | sc | 1.34+0.86 x10^11^ | 5 |
|  | ss | 4.12x10^10^ | 1 |
| **NNPLPQR** | sc | 2.18+0.84 x10^11^ | 3 |
|  | ss | 7.46x10^10^ | 1 |

sc=self-complementary vector genome conformation; ss=single-stranded vector genome conformation; vg= vector genome containing particles

**Supplementary Table S2. Transduction efficiency of indicated scAAV vector preparations**

| **Vector** | **Genomic titers**  **(x10^11^vg/ml)** | **Transducing titers**  **(x10^10^ip/ml)** | **Transduction efficiency** |
| --- | --- | --- | --- |
| **AAV2** | 1.07 | 3.21 | 3.33 |
| **VSSTSPR** | 3.46 | 8.97 | 3.86 |
| **ISSSTAR** | 2.01 | 7.04 | 2.86 |
| **NNPLPQR** | 1.69 | 3.36 | 5.02 |

vg= vector genome containing particles; ip=infectious particles; transduction efficiency=ratio of genomic/transducing titer

**Supplementary Table S3. LightCycler®96 System program for high resolution native dot blot assay.**

| **Program** | **Cycles** |
| --- | --- |
| 3 step amplification | 2 |
| 37°C for 10s |  |
| Gradient 50°C-70°C for 900s |  |
| 37°C for 10s |  |
| Cooling | 1 |
| 37°C for 30s |  |

| **Columns** | **Temperatures (°C)** |
| --- | --- |
| 1 | 50.0 |
| 2 | 51.0 |
| 3 | 52.9 |
| 4 | 55.3 |
| 5 | 57.9 |
| 6 | 60.7 |
| 7 | 63.4 |
| 8 | 65.8 |
| 9 | 67.9 |
| 10 | 69.3 |
| 11 | 69.9 |
| 12 | 70.0 |

**References**

1 Perabo, L. *et al.* In vitro selection of viral vectors with modified tropism: the adeno-associated virus display. *Mol Ther* **8**, 151-157 (2003).

2 Francois, A. *et al.* Accurate Titration of Infectious AAV Particles Requires Measurement of Biologically Active Vector Genomes and Suitable Controls. *Mol Ther Methods Clin Dev* **10**, 223-236, doi:10.1016/j.omtm.2018.07.004 (2018).

3 Wieland, S. F., Spangenberg, H. C., Thimme, R., Purcell, R. H. & Chisari, F. V. Expansion and contraction of the hepatitis B virus transcriptional template in infected chimpanzees. *Proc Natl Acad Sci U S A* **101**, 2129-2134, doi:10.1073/pnas.0308478100 (2004).
